# Regulation of thalamocortical axon branching by BDNF and synaptic vesicle cycling

**DOI:** 10.3389/fncir.2013.00202

**Published:** 2013-12-20

**Authors:** Björn Granseth, Yuichi Fukushima, Noriuki Sugo, Leon Lagnado, Nobuhiko Yamamoto

**Affiliations:** ^1^Neuroscience Laboratories, Graduate School of Frontier Biosciences, Osaka UniversitySuita, Osaka, Japan; ^2^Division of Cell Biology, Department of Clinical and Experimental Medicine, Linköping UniversityLinköping, Sweden; ^3^Sussex Neuroscience, School of Life Sciences, University of SussexBrighton, UK

**Keywords:** axon, branching, neurotrophin, synapse, endocytosis, thalamus, neocortex, development

## Abstract

During development, axons form branches in response to extracellular molecules. Little is known about the underlying molecular mechanisms. Here, we investigate how neurotrophin-induced axon branching is related to synaptic vesicle cycling for thalamocortical axons. The exogenous application of brain-derived neurotrophic factor (BDNF) markedly increased axon branching in thalamocortical co-cultures, while removal of endogenous BDNF reduced branching. Over-expression of a C-terminal fragment of AP180 that inhibits clathrin-mediated endocytosis affected the laminar distribution and the number of branch points. A dominant-negative synaptotagmin mutant that selectively targets synaptic vesicle cycling, strongly suppressed axon branching. Moreover, axons expressing the mutant synaptotagmin were resistant to the branch-promoting effect of BDNF. These results suggest that synaptic vesicle cycling might regulate BDNF induced branching during the development of the axonal arbor.

## INTRODUCTION

During development, axons form elaborate arbors to make synaptic contacts with their target cells. Neurotrophins, such as brain-derived neurotrophic factor (BDNF), have been shown to regulate axon branching in the developing brain (Cohen et al., 1954; Vicario-Abejón et al., 1998; Cohen-Cory, 1999; Marshak et al., 2007). Neurotrophins secreted from cells in a given region of the brain bind Trk or p75 receptors and activate intracellular signaling pathways in neurons projecting to that region (Chao and Hempstead, 1995). Activated Trk receptors can be internalized at the terminals and undergo retrograde trafficking along the axon via signaling endosomes (for a recent review, see Ascano et al., 2012). Growing evidence suggests that activated Trk receptors recruit different signaling pathways after endocytosis compared to when they remain on the cell surface (Grimes et al., 1996; Watson et al., 2001; Zheng et al., 2008).

Experimental evidence suggests that Trk receptor internalization is achieved through clathrin-mediated endocytosis, as Trk receptors co-localize with clathrin in immunohistochemical analyses (Grimes et al., 1996), BDNF induces the translocation of clathrin and clathrin-related proteins to the plasma membrane (Beattie et al., 2000), and RNAi targeting proteins required for clathrin-mediated endocytosis inhibit BDNF-induced retrograde signaling (Zheng et al., 2008). However, a clathrin-independent endocytic pathway has also been identified (Valdez et al., 2005). Synaptic vesicles are normally endocytosed using clathrin (Granseth et al., 2006; Zhu et al., 2009). Interestingly, Trk receptors and synaptic vesicle markers co-localize at synapses and in endosomes in dissociated cultures of neocortical neurons (Gomes et al., 2006). This indicates that clathrin-dependent endocytosis might be important for axon development.

The aim of this work is to determine if BDNF affects axon branching in the thalamocortical projection and to investigate whether synaptic vesicle cycling might influence this component of development. We demonstrate that exogenously applied and endogenously produced BDNF promote axon arbors in a co-culture system containing explants from the thalamus and cortex (Yamamoto et al., 1989). When clathrin function was reduced, the effect on branching was multifaceted; however, there were indications that endocytosis contributes to branching and synapse formation. When synaptic vesicle cycling was inhibited, branching was markedly reduced and was unresponsive to the branch-promoting effect of BDNF. These results suggest that BDNF and synaptic vesicle cycling are involved in thalamocortical axon branching.

## MATERIALS AND METHODS

### CELL CULTURE

Thalamocortical slice co-cultures were prepared according to Yamamoto et al. (1989, 1992). Cortical slices were dissected from postnatal day (P) 1 Sprague-Dawley rat pups of either sex. Dorsal thalamic blocks were prepared from embryonic day (E) 15 embryos. The thalamic and cortical slices were plated on a membrane filter (Millicell-CM PICMORG50; Millipore) coated with rat-tail collagen. The culture medium is comprised of a 1:1 mixture of Dulbecco’s modified eagle medium (DMEM) and Ham’s F-12 (Invitrogen) containing N2 supplement and 5% Fetal Bovine Serum.

Dissociated thalamic cell cultures were prepared as described previously (Maruyama et al., 2008). Thalamic blocks were prepared from E15 rat embryos. After trituration with 0.1% trypsin containing Calcium-, Magnesium-free Hanks’ solution, the cells were plated at 5000 cells/cm^2^ in 24-well culture dishes. The culture medium comprised a 1:1 mixture of DMEM and Ham’s F-12 supplemented with B27 (Invitrogen).

Dissociated hippocampal cell cultures were prepared according to Granseth et al. (2006). Hippocampi were dissected from E18 embryos, and the cells were dissociated using papain (10 U/ml). The cells were plated on 16-mm borosilicate glass coverslips at 15000–20000 cells/cm^2^ to obtain low-density neuronal networks on astrocyte monolayers. The culture medium was initially HEPES-buffered minimum essential medium (MEM) without phenol red, supplemented with N2, and 10% horse serum, but was subsequently changed to Neurobasal media without phenol red (Invitrogen), supplemented with L-glutamine and B27 at 10 days *in vitro* (DIV).

The cultures were maintained at 37°C in an environment of humidified 95% air and 5% CO_2_. All procedures were performed according to the guidelines of the animal welfare committees of Osaka University (Japan) or the Home Office regulations (UK).

### PROTEIN APPLICATION

Brain-derived neurotrophic factor (Alomone Labs) was applied at 200 ng/ml to the culture medium between 7–14 DIV. A recombinant fragment of the ligand-binding domain of the TrkB receptor fused to the Fc region of human IgG (TrkB.Fc, R&D systems) or the Fc region alone was applied at 1 μg/ml to the culture medium between 7 and 14 DIV.

### Cy3-BDNF LOADING

To produce Cy3-conjugated BDNF, 20 μl of a 32 μM BDNF (a generous gift from Sumitomo Seiyaku) solution was incubated with 0.2 μl of a 32 mM Cy3 maleimide (Amersham) solution overnight on ice. The reaction was stopped with 1 μl of 100 mM DTT. To remove free-Cy3 maleimide, the solution was passed through a gel filtration column (AutoSeq G-50, Amersham). The eluate containing Cy3-labeled BDNF was collected and confirmed using SDS-PAGE. The labeled BDNF was added to melted agar at 42°C to a final concentration of 500 μM and rapidly cooled to room temperature. Strips approximately 1 mm× 0.5 mm× 0.5 mm in size were cut and placed in the center of the cortical explant after 10 DIV.

### REVERSE TRANSCRIPTION PCR

Total RNA was extracted from thalamic explants, and cDNA was synthesized. A DNA fragment (174 bp) of *trkB* (NM_001163168) was amplified by PCR with a pair of primers (5′-TCTCCAGGAGACGAAATCCAGCC-3′ and 5′-CTGCAGGAAATGGTCACAGA-3′). The cycling parameters were 32 cycles at 95°C (30 s), 55°C (20 s), and 72°C (2 min).

### PLASMID CONSTRUCTION

The coding region of a fusion protein of the C-terminal fragment of accessory protein 180 (AP180C) and monomeric red fluorescent protein (mRFP) was cloned into a pCAGGS vector (Niwa et al., 1991; Granseth et al., 2006) or the pTRE-Tight response vector of the Tet-On Advanced gene expression system (Clontech). To optimize the Tet-On Advanced plasmid for use in the slice culture system, the coding region for the reverse tetracycline-controlled transactivator protein (rtTA^2^M2) was cloned into the pCAGGS vector. No mRFP-AP180C production could be detected through fluorescence microscopy in cells double transfected with pCAGGS-rtA^2^M2 and pTRE-mRFP-AP180C until doxycycline was added to the culture medium at 12 DIV. The control cells expressed enhanced green fluorescent protein (EGFP) from the pCAGGS vector.

To prepare the synaptotagmin expression plasmids, the coding region for wild-type synaptotagmin 1 (Syt1) or mutant Syt1 (mSyt1) was cloned into an expression vector. Total RNA was extracted from P2 rat brain RNA, and was subjected to reverse transcription (Thermoscript RT-PCR system, Invitrogen). To obtain Syt1 cDNA (Genbank: AJ617615), PCR was carried out with a set of primers (5′-ATCCGCAGTCAGATCGGAAG-3′ and 5′-AAGAGCACTATGTGGGCAGA-3′). The obtained cDNA was subcloned into pGEM-T vector (Promega), and the cDNA containing the coding region was further amplified with primers containing *XhoI* site (5′-GCTCGAGATGGTGAGTGCCAGTCATCC-3′ and 5′-CGGATCCTTCTTGACAGCCAGCATGG-3′) to be cloned into a pCAGGS (Niwa et al., 1991) or pCMV plasmid. To generate the mSyt1 expression plasmid, a Ca^2^^+^-binding aspartic acid at position 209 was substituted with asparagine (Nishiki and Augustine, 2004). For this, the whole pCAGGS-Syt1 was subjected to PCR with two complementary primers (5′-GTGGGTGGCTTATCTAATCCCTACGTGAAG-3′ and 5′-CTTCACGTAGGGATTAGATAAGCCACCCAC-3′) containing a mutation site (underlined), which produces the amino acid replacement.

### TRANSFECTION

To visualize thalamic axons in thalamocortical slice co-cultures, an expression plasmid (pCAGGS) encoding EGFP or enhanced yellow fluorescent protein (EYFP) was transfected into a small number of thalamic neurons at 1 DIV using an electroporation method as thoroughly described in Uesaka et al. (2005, 2008). The plasmid solution was applied through a fire-polished borosilicate glass micropipette (50-μm tip diameter), and electrical pulses (five to seven trains of 200 square pulses of 1 ms duration at 200 Hz, 500–700 μA) were delivered through a second borosilicate micropipette (tip diameter of 200–300 μm). Two to four sites were electroporated on each thalamic explant. The plasmids, pCAGGS-mSyt1 and pCAGGS-Syt1 were co-transfected with either pCAGGS-EGFP or pCAGGS-EYFP. The plasmid concentrations used were 2.0 and 1.0 μg/μl for pCAGGS-mSyt1/Syt1 and pCAGGS-EGFP/EYFP, respectively. Electroporations using the Tet-On system were performed with a plasmid solution containing pCAGGS-rtA^2^M2, pTRE-mRFP-AP180C, and pCAGGS-EGFP at 2.0, 2.0, and 1.0 μg/μl, respectively.

Transfections in dissociated cell culture were performed using Lipofectamine 2000 (Invitrogen) according to the manufacturer’s instruction. The hippocampal cells were transfected at 12 DIV with pCMV-synaptophysin-pHluorin (SypHy) and pCMV-mRFP. The thalamic cells were transfected with pCMV-mSyt1 and pCMV-EGFP immediately before plating the cells.

### IMMUNOHISTOCHEMISTRY

After 14 DIV, the slice cultures were fixed with 4% paraformaldehyde in phosphate buffered saline (PBS) on ice for 1 h and pre-incubated in blocking solution containing 20% normal goat serum and 0.3% Triton X-100 in PBS at room temperature for 3 h. The cultures with EGFP or EYFP labeled axons were incubated with rat monoclonal anti-GFP (1:2000; Nacalai Tesque) at 4°C for 24 h followed by an Alexa488-conjugated anti-rat secondary antibody (1:400, Invitrogen) at 4°C for 12 h. For double labeling of EGPF and mRFP, the cultures were incubated for an additional 24 h at 4°C with rabbit polyclonal anti-RFP (1:2000, Medical and Biological Laboratories Co.) followed by Cy3-conjugated anti-rabbit (1:400; Chemicon) at 4°C for 12 h. The slices were mounted on glass slides using a 50% glycerol mounting medium containing 2.3% 1,4-diazobicyclo[2.2.2]octane (DABCO, Sigma Aldrich), cover-slipped and sealed with nail polish.

### MICROSCOPY

Alexa488-labeled axons were visualized through confocal microscopy at a >515 nm fluorescence emission excited at 488 nm using an argon-laser scanning microscope (MRC-600; Bio-Rad). Images were obtained using a 10×, 0.30 NA objective and digitized at a depth of 8 bits. For the Alexa488/Cy3 double-labeled cultures, the fluorescence was sequentially detected for two channels using a Zeiss LSM 700 to avoid emission spectral bleed-through. For Alexa488, a 495–544 nm fluorescence emission from 488 nm diode laser excitation was used. For Cy3, a 560-nm longpass fluorescence emission from 555 nm diode laser excitation was used. Confocal stacks were captured using 10×, 0.45 NA and 40×, 1.3 NA Zeiss objective lenses and digitized at a depth of 16 bits. Overlapping image stacks consisting of 2–20 optical sections 1–5 μm apart were collected to completely visualize the entire axon arbor. Individual optical sections were normalized according to average background fluorescence and collapsed to a single plane using the maximum intensity for each pixel. Axons were reconstructed using the NeuronJ plugin for ImageJ (Meijering et al., 2004). An analysis of the axonal tracings was performed using IgorPro (Wavemetrics). Small processes (<5 μm) were excluded from analysis. The axonal fluorescence intensity was calculated by subtracting the background fluorescence measured using an offset version of the axon trace from the average intensity for pixels along the axon.

### SYNAPTIC VESICLE IMAGING

Hippocampal cultures were imaged at 14 DIV using a Photometrics Cascade 512B camera mounted on an inverted Nikon Diaphot 200 microscope with a 40×, 1.3 NA, oil immersion objective. Images were captured at a depth of 16 bits. The cells were superfused with a pH 7.4 buffer containing (in mM) 136 NaCl, 2.5 KCl, 10 HEPES, 1.3 MgCl_2_, 10 glucose, 2 CaCl_2_, 0.01 CNQX, and 0.05 dl-APV. All chemicals were obtained from Sigma Aldrich, and the receptor antagonists were purchased from Tocris Cookson. A 100 W Xenon arc lamp was used for illumination. To minimize photo-bleaching, the light was attenuated 4–8 times using neutral density filters. A Uniblitz VMM-D3 shutter was used to restrict illumination to periods when the camera was actively acquiring images. Action potentials were evoked through field stimulation (20 mA, 1 ms pulses) in a custom-built chamber with two parallel platinum wires 5 mm apart (Royle et al., 2008). The image sequences were imported into IgorPro (Wavemetrics) and analyzed using custom-written scripts. Square regions of interest (ROIs) measuring 4.8 μm × 4.8 μm were positioned on synapses identified by the presence of a >2 SD SypHy fluorescence increase to 40 APs at 20 Hz compared with the baseline noise. The SypHy fluorescence was quantified using a 475:40 nm bandpass excitation filter, a 505 nm dichroic mirror, and a 535:45 nm bandpass emission filter (Omega Filters). For mRFP-AP180C, the filterset comprised a 560:35 nm bandpass excitation filter, a 595 nm dichroic mirror, and a 645:75 nm bandpass emission filter. To subtract the local background fluorescence, the intensity of the ROI displaced in the x- or y-direction was used (Royle et al., 2008). A corrected baseline for individual synapses prior to averaging was used to normalize the traces. All traces were visually inspected before averaging.

### STATISTICS

The data are presented as the means ± standard error of the mean (SEM). A value of *P* < 0.05 was considered statistically significant using Student’s *t*-test unless otherwise indicated. Multiple comparisons were evaluated using analysis of variance (one-way ANOVA) with a significance level 0.05 with *post hoc* Newman–Keuls test. The Kolmogorov–Smirnov test was used to compare cumulative distributions. All analyses were performed using IgorPro.

## RESULTS

### BDNF PROMOTES THALAMOCORTICAL AXON BRANCHING

**FIGURE 1:**
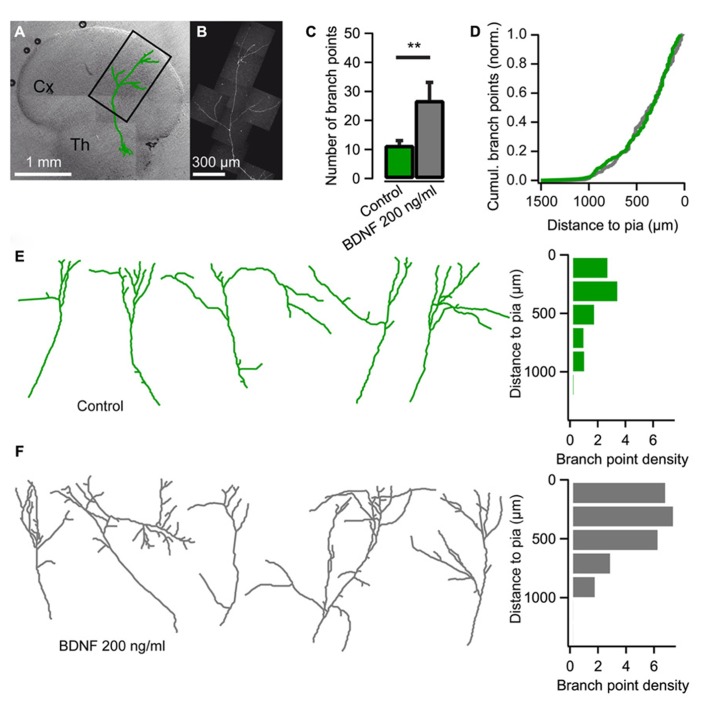
**Brain-derived neurotrophic factor (BDNF) promotes axon branching in thalamocortical co-cultures. (A)** Reconstruction of a thalamic neuron expressing EYFP (green) superimposed on a differential interference contrast-enhanced micrograph of a thalamocortical co-culture. *Cx* cerebral cortex; *Th* thalamus. **(B)** Confocal micrograph of the axon from the neuron reconstructed in A. **(C)** Bar diagram for the average number of axonal branch points (the number of times the axon bifurcates) when BDNF is added to the medium. The error bars represent SEM. **P < 0.01. **(D)** Cumulative plot of branch-point location with respect to pial surface for control cultures (green) and cultures treated with BDNF (gray). **(E)** Reconstructions of representative axons from thalamic cells in the neocortical explant and a histogram showing the distribution of axonal branch points with respect to the pial surface. Branch-point density = number of branch points/number of axons. **(F)** Reconstructions of representative axons from thalamic cells in the neocortical explant when BDNF was added to the medium. A histogram plotting the distribution of the axonal branch points with respect to the pial surface is shown on the right.

Using co-cultures of the thalamus and cortex (Yamamoto et al., 1989) we set out to investigate the effect of BDNF on thalamocortical axon branching. Low numbers of thalamic cells were transfected with EYFP or EGFP encoding plasmids so individual axons could be observed in the neocortical explant (**Figures 1A,B**). As demonstrated previously, thalamocortical axons branched extensively in the neocortical explant after 14 DIV (**Figure 1E**; Yamamoto et al., 1992; Uesaka et al., 2007). The average number of branch points was 11.4 ± 1.6 (*n* = 19; **Figure 1C**), and consistent with previous studies (Yamamoto et al., 1992; Uesaka et al., 2007), the majority of branches were formed in the upper layers of the cortical explant (**Figures 1D,E**).

When BDNF (200 ng/ml) was added to the culture medium at 10 DIV, thalamic axon branching was substantially increased (**Figure 1F**). At 14 DIV, the number of branch points more than doubled to 26.9 ± 6.2 when BDNF was present (*n* = 9, *P* < 0.01; **Figure 1C**). The laminar location of the BDNF-induced branches seemed normal since the cumulative plot of branch point distribution across the cortical layers was not significantly different from control condition (**Figure 1D**; Kolmogorov–Smirnov test). Thus, BDNF promotes axonal branching without disturbing the laminar pattern of branch formation.

**FIGURE 2:**
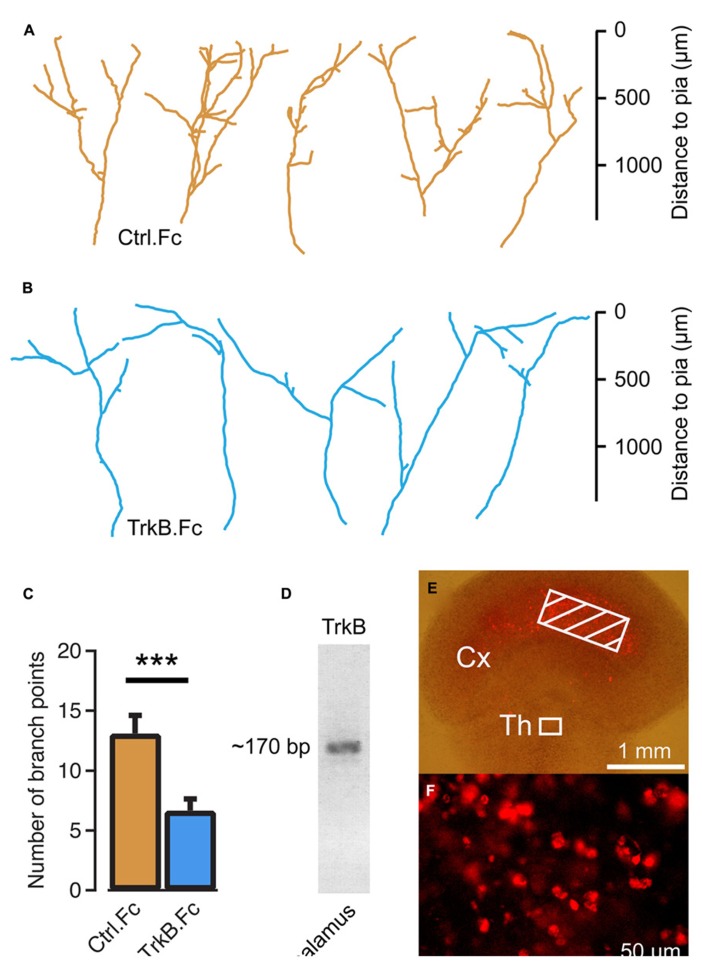
**Endogenous brain-derived neurotrophic factor (BDNF) supports thalamocortical axon branching. (A)** Reconstructions of representative axons in thalamocortical co-cultures treated with the Fc region of human IgG. Note the similar morphology to untreated controls in **Figure 1E**. **(B)** Reconstructions of representative thalamocortical axons when TrkB.Fc was added to the culture to remove endogenously produced BDNF. **(C)** Bar diagram for average number of branch points for TrkB.Fc treatment and Fc controls. The error bars represent SEM. ***P < 0.001. **(D)** Reverse transcriptase PCS product from thalamic explants in co-cultures. The ~170 bp PCR product suggests that thalamic cells produce TrkB. **(E)** BDNF tagged with Cy3 was molded in strips of agar and applied to the neocortical explant (hatched region) in thalamocortical co-cultures. **(F)** Neurons in the thalamic explants contained Cy3-BDNF 5 days later. Fluorescence micrograph from the boxed region in E.

To investigate the role of endogenous BDNF, TrkB.Fc was added to the culture medium at 10 DIV to sequester the existing neurotrophin. We found that branch formation of thalamocortical axons was inhibited in the presence of TrkB.Fc, whereas axons grew up to the pial surface. The number of axonal branch points at 14 DIV was significantly reduced in the presence of TrkB.Fc (6.23 ± 0.91, *n* = 17, *P* < 0.001; **Figures 2B,C**) compared to the control condition when the Fc without TrkB was used (13.1 ± 1.49, *n* = 18; **Figures 2A,C**). Reverse transcription PCR on thalamic explants revealed that *trkB* was expressed in the cultured thalamic cells (**Figure 2D**), indicating that exogenous and endogenous BDNF can promote thalamocortical axon branching via TrkB receptors.

We further investigated whether exogenous BDNF may be taken up from thalamocortical axon terminals. To present neurotrophin to the thalamic axons at a site remote from the cell bodies and their dendrites, agar strips containing 500 μM of Cy3-tagged BDNF were placed on the neocortical explant side at a distance of >1 mm from the thalamic explant (**Figure 2E**). After a 5-day incubation, Cy3-BDNF fluorescence was present at the soma of thalamic neurons (**Figure 2F**). It appears that the labeled BDNF was taken up by the terminals and transported retrogradely to the thalamus.

### CLATHRIN-MEDIATED ENDOCYTOSIS IN THALAMOCORTICAL AXONS

**FIGURE 3:**
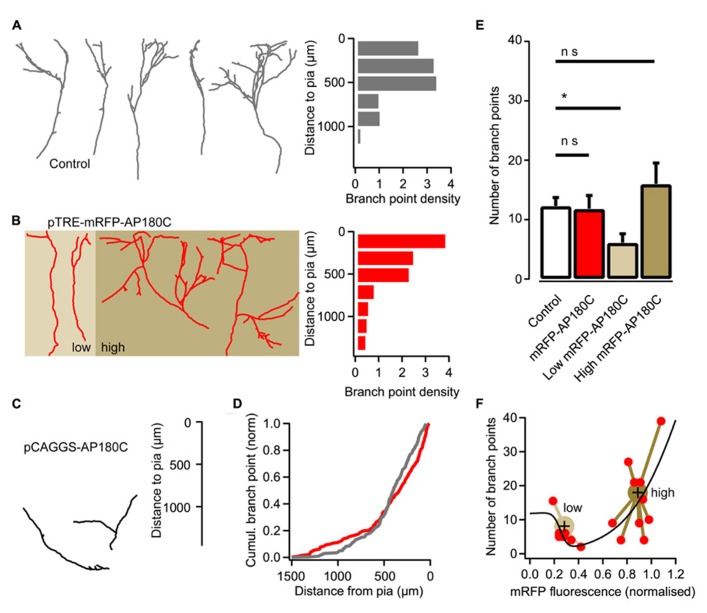
**Reducing clathrin-mediated endocytosis with AP180C affects axon branching. (A)** Reconstructions of representative axons from thalamic cells in the neocortical explant and a histogram showing the distribution of axonal branch points with respect to the pial surface. Control axons exposed to doxycycline for two days. Branch-point density = number of branch points/number of axons. **(B)** Reconstructions of representative axons from thalamic cells where the expression of mRFP-AP180C was induced with doxycycline two days earlier. A histogram showing the distribution of axonal branch points with respect to the pial surface is shown on the right. Axons expressing low amount of mRFP-AP180C are against a light brown background. Axons expressing high amount of the construct are against a dark brown background. **(C)** Reconstructions of representative axons from thalamic cells expressing mRFP-AP180C for 13 days. **(D)** Cumulative plot of the branch-point location with respect to the pial surface for doxycycline-induced mRFP-AP180C-expressing axons (red) and controls (gray). **(E)** Average number of branch points for doxycycline-exposed controls and induced mRFP-AP180C-expressing cells. When mRFP-AP180C-expressing cells were divided into two groups according to a k-means clustering algorithm (see panel **F**), the group with low mRFP-AP180C fluorescence had fewer branch points than the controls. The error bars represent SEM. *P < 0.05 (ANOVA). **(F)** Number of branch points plotted against mRFP-AP180C fluorescence intensity. The black line illustrates opposing effects on branch number from two AP180C-mediated mechanisms using the sum of two sigmoid functions with opposite signs and parameters fit using least sum of squares. Large markers are centroids from a k-means clustering analysis with the relevant data points connected with lines.

Several studies support clathrin-mediated endocytosis as the mechanism for internalizing BDNF bound to TrkB receptors (Grimes et al., 1996; Beattie et al., 2000; Zheng et al., 2008). Thus, we examined whether a clathrin-dependent endocytic mechanism is important for thalamic axon branching. Clathrin-mediated endocytosis can be inhibited through the over-expression of AP180C, which contains several clathrin- and adaptor protein 2 (AP2)-binding domains (Ford et al., 2001; Granseth et al., 2006). However, when AP180C was introduced into thalamic cells through electroporation at the second day *in vitro*, the axons did not extend their axons more than halfway into the neocortical explant at 14 DIV (**Figure 3C**). The AP180C transfection seemingly resulted in a defect in initial axonal growth.

To induce the expression at the appropriate point in time when thalamic axons are forming branches after reaching the target layer, an inducible Tet-On expression system was used. For this, thalamocortical neurons were transfected with pCAGGS-rtA^2^M2, pTRE-mRFP-AP180C and pCAGGS-EGFP (see Materials and Methods) at 1–2 DIV, and doxycycline was added to the culture medium at 12 DIV. When axon branching was examined two days later, the number of branch points (11.8 ± 2.2, *n* = 19) was not significantly different from control axons (12.3 ± 1.4, *n* = 19; **Figures 3A,B,E**). However, the location of branches was affected (**Figure 3D**; *P* < 0.05, Kolmogorov–Smirnov test). More branch points were present in the deeper and the most superficial layer of the cortical explant, and less branch points were found in the appropriate laminae (**Figure 3B**).

The mislocalization of branch points in AP180C-overexpressing thalamic axons could be from defects in clathrin-mediated endocytosis as well as other clathrin-mediated cellular mechanisms. The C-terminal fragment of AP180C contains AP2- and clathrin-binding domains but lacks the membrane-binding N-terminal domain (Ford et al., 2001; Zhao et al., 2001). Thus, over-expression could have two molecular consequences: (1) competitive antagonism of endogenous AP180 when clathrin is recruited to AP2 during endocytosis, and (2) clathrin sequestration in the cytosol that diminish the availability of this molecule for endocytosis and other clathrin-mediated cellular processes (Zhao et al., 2001). The first mechanism would be specific for endocytosis, while the second mechanism would affect all clathrin-mediated cellular processes in the neuron. Unassembled clathrin constitutes as much as 0.1% of the total cell-protein mass (Goud et al., 1985); therefore, clathrin-sequestration requires a high concentration of AP180C. Since the AP180C is tagged with mRFP we can get an estimate of intracellular concentration by measuring fluorescence intensity in maximum-intensity z projections of confocal-image stacks. To examine if the two mechanisms have different dose response curves we fitted the sum of two sigmoid curves for branch number as a function of fluorescence intensity (**Figure 3F**). When expression of mRFP-AP180C was low, the number of branches was diminished, and with increasing concentration the number of branch points increased. A k-means clustering algorithm sorted the data into two groups (**Figure 3F**). Neurons with low levels of mRFP-AP180C had fewer branch points than the controls (*P* < 0.05, ANOVA, *n* = 8; **Figure 3E**). The number of branch points in neurons with high expression of mRFP-AP180C was not significantly different to controls (ANOVA, *n* = 11; **Figure 3E**) although branches were often mislocalized (*P* < 0.05, Kolmogorov–Smirnov test). Because of the increased number of mislocalized branches, it seems reasonable to infer that branching in the right cortical laminae might be reduced also in neurons expressing high levels of AP180C.

**FIGURE 4:**
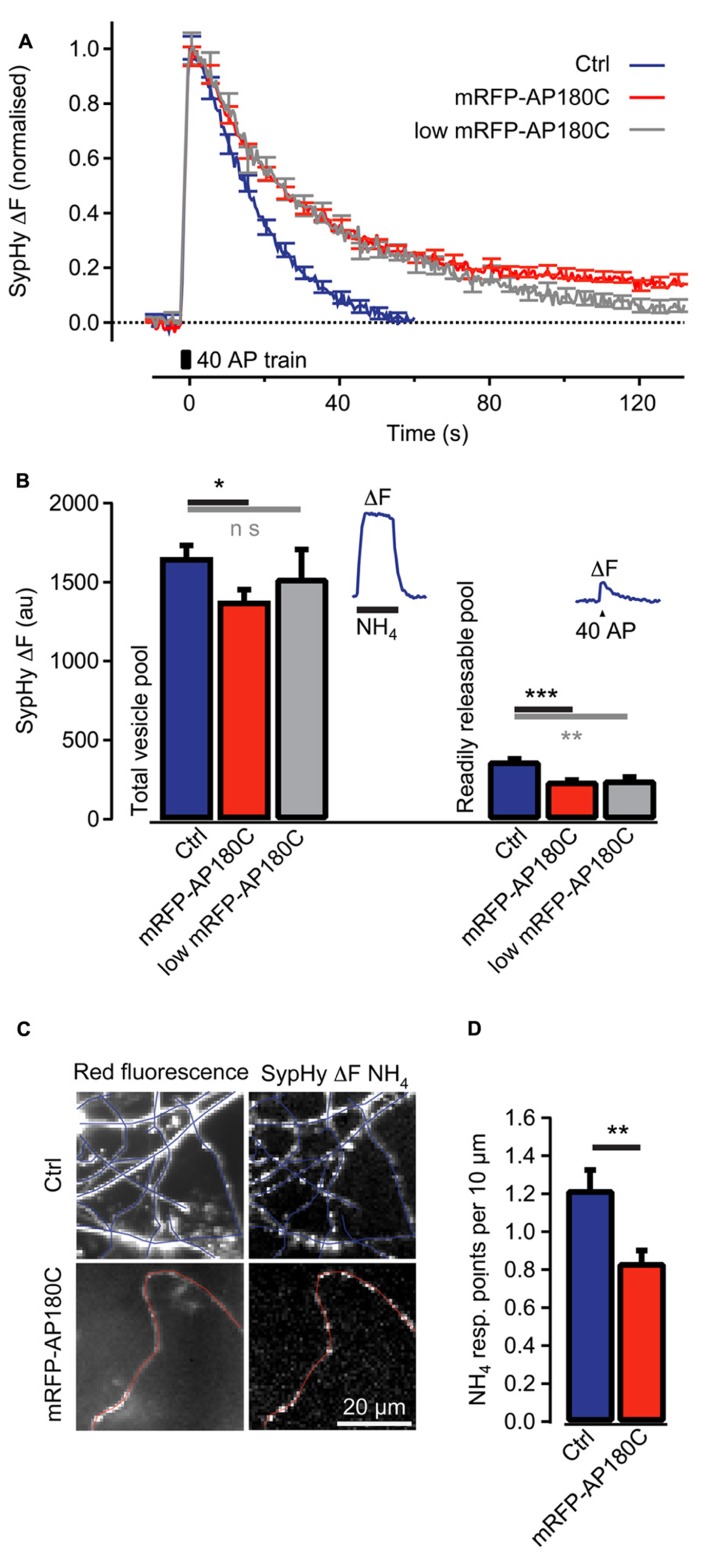
**Clathrin-mediated endocytosis of synaptic vesicle membrane is reduced in synapses even when mRFP-AP180C content is low. (A)** Change in SypHy fluorescence intensity (normalized to the peak) after 40 action potentials at 20 Hz (40 AP train). During the action potential train, the fluorescence intensity increased from exocytosis of synaptic vesicles when SypHy in the acidic lumen of vesicles was exposed to the neutral pH of the extracellular space. After termination of the action potential train, the fluorescence returned to baseline when SypHy was endocytosed with the vesicle membrane and the vesicle lumen re-acidified. The blue trace indicates control synapses with no detectable mRFP-AP180C fluorescence. The red line indicates entire population of synapses containing mRFP-AP180C. The gray trace indicates the 20% of synapses with the least intense mRFP-AP180C fluorescence. **(B)** Total vesicle pool estimated through the fluorescence change from collapsing the pH of all synaptic vesicles using an NH_4_-containing buffer at pH 7.4. Readily releasable pool estimated from the fluorescence change after 40 action potentials at 20 Hz. The error bars represent SEM. *P < 0.05, **P < 0.01, ***P < 0.001. The inset shows the average response for control synapses in one culture to illustrate the protocol. **(C)** Micrographs of red fluorescence in control axons (Ctrl) expressing mRFP and axons expressing mRFP-AP180C and the increase in green fluorescence (ΔF) from SypHy, induced by NH_4_-containing buffer. Blue and red lines are tracings along the axons. **(D)** Average number of points per 10 μm of axon that responds to NH_4_-containing buffer. These points where SypHy containing vesicles are accumulated are the locations of putative synapses. The error bars represent SEM. *P < 0.05, **P < 0.01, ***P < 0.001.

The sequestration of intracellular clathrin at high concentration of AP180C has been demonstrated using immunohistochemistry (Zhao et al., 2001). An additional mode of action that requires less AP180C, such as the competitive antagonism of endogenous AP180, has not yet been demonstrated in living cells. To investigate if AP180C could act in this way, we investigated if the clathrin-mediated endocytosis of synaptic vesicles was affected when the molecule was present at low concentration. Neurons in hippocampal cultures were double-transfected using vectors for mRFP-AP180C and SypHy (Granseth et al., 2006; Zhu et al., 2009). The cells were incubated for less than 48 h for expression, and the synapses with the lowest mRFP-AP180C fluorescence intensity were selected for separate analysis (20% of the total pool). The speed of clathrin-mediated endocytosis, as measured using the vesicle cycling probe SypHy, was markedly reduced compared with that of the controls (**Figure 4A**). The slowing of the vesicle membrane uptake in the 20% of synapses with the lowest mRFP-AP180C expression was not substantially different from that seen for the entire mRFP-AP180C expressing group in general (**Figure 4A**). Although there was no statistically significant reduction in the total number of synaptic vesicles in synapses with low expression when measured by NH_4_ dequenching (*n* = 42), the readily releasable pool of vesicles, that is, the number of vesicles released after 40 action potentials at 20 Hz, was markedly reduced (*P* < 0.01; **Figure 4B**). Thus, clathrin-mediated endocytosis is impaired in the 20% of synapses with mRFP-AP180C levels just above our detection limit for live fluorescence imaging (>2 SEM of background). This result is consistent with the possibility that AP180C acts as an antagonist to endogenous AP180, supporting the interpretation that the reduction of axon branches at low AP180C expression level results from inhibition of clathrin-mediated endocytosis.

Axon branching is closely associated with the formation of synapses (Alsina et al., 2001; Ruthazer et al., 2006; Fukunishi et al., 2011). We examined the varicosities that are the likely locations of synapses along the axons in the hippocampal cultures. SypHy fluorescence responses to NH_4_ were usually localized to these varicosities, indicating that they contain large numbers of labeled synaptic vesicles (**Figure 4C**). Axons expressing mRFP-AP180C had fewer of these putative synapses per unit length of axon (0.84 ± 0.07 synapses/10 μm, *n* = 8, **Figure 4D**) compared to controls (1.21 ± 0.10 synapses/10 μm, *n* = 9, *P* < 0.01). The smaller number of putative synapses in AP180C-expressing axons in hippocampal culture is in accordance with the decreased number of axonal branches in thalamocortical co-cultures.

### SYNAPTIC VESICLE CYCLING AND BDNF SIGNALING

**FIGURE 5:**
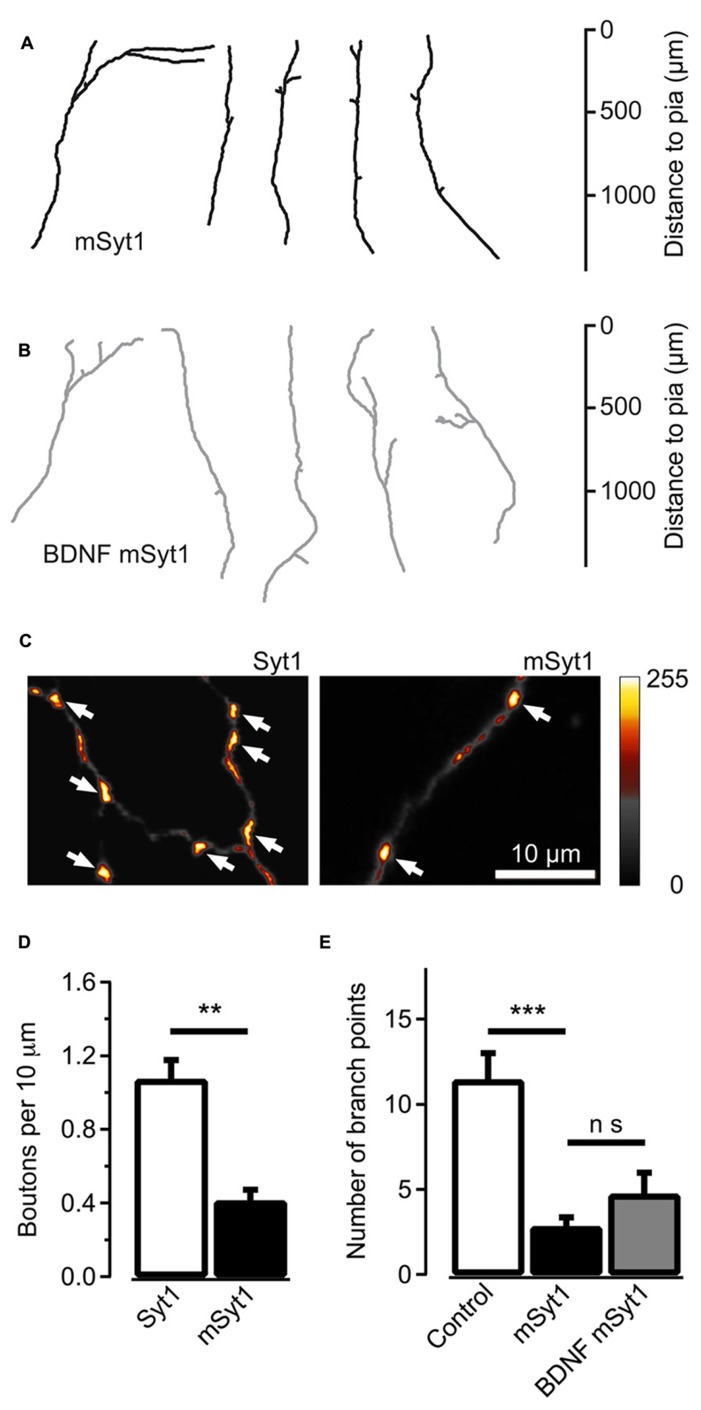
**Reducing synaptic vesicle cycling produces less axonal branches. (A)** Reconstructed axons of thalamic neurons expressing mSyt1. **(B)** Reconstructed axons of mSyt1-expressing neurons supplemented with 200 ng/ml of brain-derived neurotrophic factor (BDNF). Note the similar morphology to the axons without BDNF. **(C)** Pseudocolor micrographs of axons expressing wild type Syt1 or mSyt1. The arrows indicate the locations of varicosities where the EYFP fluorescence accumulates, indicative of presynaptic boutons. **(D)** Average number of fluorescence accumulations per 10 μm of axon in the control cells and neurons expressing mSyt1. **(E)** The average number of branch points is reduced in axons expressing mSyt1 and is not significantly affected by BDNF present in the growth medium. The error bars represent SEM. **P < 0.01, ***P < 0.001.

The AP180C construct potentially slows clathrin-mediated endocytosis of both Trk receptors and synaptic vesicles. To selectively target synaptic vesicle cycling, we over-expressed mSyt1 where a Ca^2^^+^ binding aspartic acid (209) in the C2B domain was substituted with asparagine (Mackler et al., 2002; Nishiki and Augustine, 2004). This will reduce the exocytosis of synaptic vesicles with an accompanying reduction in endocytic activity at the terminals. The mSyt1 and EYFP expression vectors were co-electroporated into thalamic cells in thalamocortical co-cultures at 2 DIV. At 14 DIV, mSyt1-expressing thalamocortical axons showed less developed axonal arbors (average number of branch points = 2.7 ± 0.6, *n* = 19, *P* < 0.001; **Figures 5A,E**). High-resolution fluorescence microscopy showed that axonal varicosities were less frequent along axons expressing mSyt1 than axons expressing wild-type Syt1 (*P* < 0.01; **Figures 5C,D**). Fewer putative presynaptic boutons per unit length and less extensive axonal arbors suggest that the total number of synaptic connections was severely reduced in neurons with synaptic vesicles carrying the mutant Syt1 protein.

**FIGURE 6:**
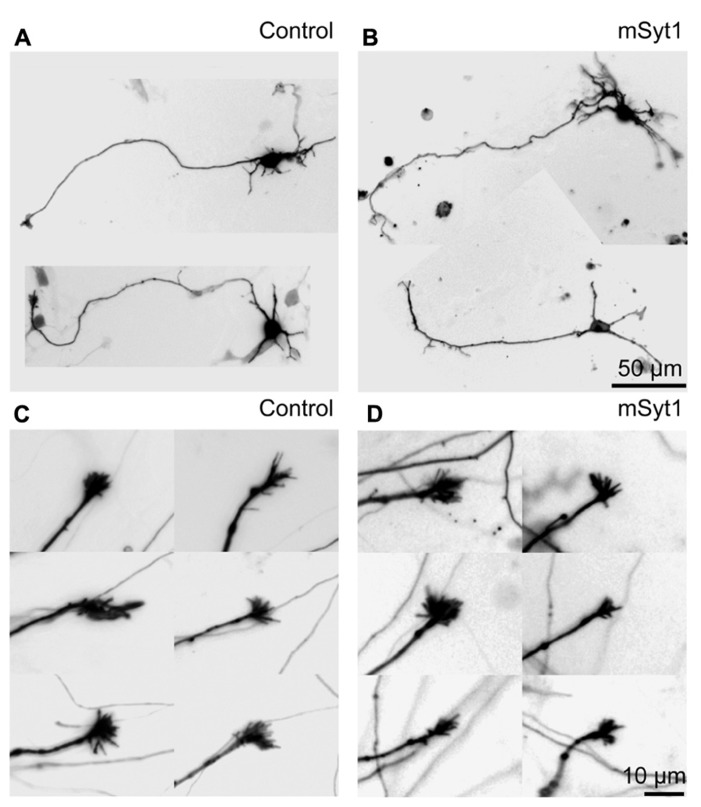
**Axon growth is normal in thalamic neurons expressing mutant synaptotagmin. (A)** Representative fluorescence micrographs of thalamic dissociated neurons in culture. The LUTs are inverted for clarity. **(B)** Representative neurons expressing mSyt1. **(C)** Representative fluorescence micrographs of axonal growth-cones of thalamic neurons in culture. **(D)** The growth cones in the axons of thalamic neurons expressing mSyt1 show a normal morphology.

It is unlikely that the small axonal arbors in mSyt1-expressing axons resulted from late-arriving axons having less time to develop branches and synapses. The mSyt1-expressing thalamocortical axons extended well into the upper layers of the neocortical explants (**Figure 5A**), which was different from the obvious growth defect associated with AP180C without the inducible vector (**Figure 3C**). To investigate whether there might be a subtle defect in axon growth when expressing mSyt1, we characterized neurite outgrowth and elongation in dissociated cell culture of E15 thalamic neurons (**Figures 6A,B**). The growth cones were not different from the controls (**Figures 6C,D**), and after 5 days in culture, neurite length of mSyt1-expressing thalamic cells (191 ± 15 μm, *n* = 32) was similar to that of the controls (176 ± 14 μm, *n* = 27). Thus, there is no reason to suspect that the mSyt1 axons arrive in the cortex any later than the controls.

To determine if the reduction in branch number in mSyt1 expressing axons could be related to BDNF signaling, we added recombinant BDNF to the culture medium at the same concentration that induced the marked expansion of axonal branch points in naïve axons (**Figure 1C**). If the effect of synaptic vesicle cycling on axonal branching is unrelated to BDNF signaling, exogenously applied neurotrophin should be efficient at increasing branch numbers. The axons expressing mSyt1 were however unresponsive to the branch-promoting effect of BDNF (**Figures 5B,E**). The average number of branch points was 4.8 ± 1.3 (*n* = 12), which was not significantly different from untreated mSyt1 axons. BDNF-treated mSyt1 neurons remained significantly smaller than untreated wild-type control axons (*P* < 0.01). Thus, inhibiting synaptic vesicle release through mSyt1 desensitizes thalamocortical axons to the branch-promoting effects of BDNF, suggesting that synaptic vesicle cycling might facilitate this aspect of BDNF signaling.

## DISCUSSION

The present study demonstrates that BDNF promotes the development of branches in thalamocortical axons. Both exogenously added BDNF and endogenously produced neurotrophin, acting on TrkB receptors in the thalamic neurons, increase axonal branching during development. Although the results from the experiments targeting clathrin function were complex, there were indications that endocytosis supports branching and synapse formation. When synaptic vesicle cycling was inhibited, branching was markedly reduced and was unresponsive to the branch-promoting effect of BDNF. We propose a hypothesis that synaptic vesicle cycling enhaces BDNF signaling via clathrin-dependent endocytosis of activated TrkB receptors.

### BRAIN-DERIVED NEUROTROPHIC FACTOR PROMOTES THALAMOCORTICAL AXON BRANCHING

Wiesel and Hubel (1963) showed that the projection from the thalamus will segregate into eye-specific columns in the primary visual cortex after eye-opening. The development of these ocular dominance columns can be inhibited by intracortical injections of BDNF (Cabelli et al., 1995; Galuske et al., 1996) or by removal of endogenous BDNF via TrkB.Fc (Cabelli et al., 1997). Moreover, BDNF is known to be involved in dendritic development of cortical neurons (McAllister et al., 1995, 1997). Until now, the effects of BDNF on thalamocortical axon branching have not been investigated. The present paper demonstrates that BDNF is a potent branch-promoting factor for these axons (**Figures 1** and **2**). BDNF has been known to act both as a retrograde factor (Du and Poo, 2004; Zweifel et al., 2005), and as an anterograde factor (Altar et al., 1997; Conner et al., 1997; Caleo et al., 2000; Kohara et al., 2001; Kojima et al., 2002). Since exogenous BDNF applied only to the cortical portion of the co-culture system can be taken up by thalamic axons and transported to the soma (**Figures 2E,F**), BDNF seems to function as a retrograde signal for the developing thalamocortical neurons.

Recently, Jeanneteau et al. (2010) identified a mechanism by which BDNF promotes branch formation in neocortical pyramidal neurons. BDNF-TrkB activation enhances MAP kinase phosphatase-1 (MKP-1) function by inducing its production and by protecting it from degradation. This phosphatase deactivates c-jun N-terminal kinase (JNK) leading to destabilization of cytoskeletal components and promoting branch formation. Interestingly, selective administration of BDNF to axons robustly induced MKP-1 expression. It is unknown if this retrograde signal might rely on endocytosis and axonal transport of TrkB signaling endosomes.

### SYNAPTIC VESICLE CYCLING SUPPORTS AXONAL ARBORIZATIONS

When an action potential reaches the presynaptic bouton, neurotransmitter is released through exocytosis of small synaptic vesicles. The vesicle membrane that has fused with the presynaptic membrane will be recycled to make new vesicles via clathrin-mediated endocytosis (Granseth et al., 2006; Zhu et al., 2009). AP180C slowed down clathrin-mediated endocytosis of synaptic vesicles, but also reduced neurotransmitter release (**Figure 4B**). This is similar to the effect on synaptic vesicle exocytosis seen in the *Drosophila* AP180 knockout (Bao et al., 2005) and when AP2 function is inhibited in the Calyx of Held (Hosoi et al., 2009). While the direct action of mSyt1 was to inhibit neurotransmitter release, it also reduces endocytosis as there is less vesicular membrane to be recycled. In the present study, both manipulations reduced thalamocortical axon branching in the target layer (**Figures 3E** and **5E**), suggesting that synaptic vesicle cycling promotes axon arbor formation.

A recent investigation of thalamocortical axon branching in a SNAP-25 knockout mouse came to a different conclusion (Blakey et al., 2012). Branching was similar in thalamocortical co-cultures when the thalamic explant was prepared from knockouts or from wild-type mice. The conflicting results might be explained by differences in the magnitude and distribution of the induced defects in vesicle cycling. Synchronous synaptic vesicle release is completely absent in the SNAP-25 knockout while mSyt1 and AP180C cause graded reductions of vesicle cycling. All neurons in the thalamic explant from the knockout mouse have the defect in transmitter release while only a small proportion of neurons express mSyt1 or AP180C after electroporation.

### A HYPOTHESIZED ROLE FOR ENDOCYTOSIS IN ACTIVITY-DEPENDENT AXON COMPETITION

The mechanism that initiates the endocytic uptake of activated Trk receptors is unknown. Since endocytosis of synaptic vesicle membrane and Trk receptors occurs at the same axonal location (Gomes et al., 2006; Granseth et al., 2006), and use the same core components for endocytic internalization (Grimes et al., 1996; Beattie et al., 2000; Granseth et al., 2006; Zheng et al., 2008; Zhu et al., 2009; McMahon and Boucrot, 2011), we would like to propose the hypothesis that Trk receptor endocytosis is enhanced by synaptic vesicle endocytosis following transmitter release. This hypothesis not only explains the experimental findings of the present study, but also provides a mechanism for activity-dependent competition and cooperation between axon arbors during development.

Neuronal activity increases the exocytic secretion of BDNF from neocortical cells (Kolarow et al., 2007; Matsuda et al., 2009). If neighboring axons compete for the supply of this retrograde factor and endocytosis of activated TrkB receptors is enhanced during a time-window defined by synaptic vesicle endocytosis, the thalamic axons that reliably deporarize the target neuron will have a competitive advantage over those that do not. The enhanced survival and growth of stronger axons over weaker axons with disparate action potential firing pattern will lead to a gradual activity-dependent refinement of innervation where relevant axons are kept while unrelated axons retract.

This hypothesis can explain the effects we observed with AP180 and mSyt1. Axons in which vesicle cycling was impaired by mSyt1 would rapidly fail in the competition against normal axons, leading to the markedly reduced axonal arbors we obtained in the present study (**Figure 5E**). Axons with slowed clathrin-mediated endocytosis produced by AP180C would also be at a competitive disadvantage because the mechanism for Trk receptor internalization is impaired. However, since synaptic vesicle endocytosis is slowed-down, the time window during which Trk receptor internalization is enhanced by synaptic activity might be increased. Thus, the effect on branching might be less pronounced, as we observed in the present investigation (**Figure 3E**).

In the proposed hypothesis, AP180C and mSyt1 reduce axon branching through their effects on clathrin-mediated endocytosis. An alternative hypothesis would be that they inhibit branching through their effect on exocytosis. Branching and synapse formation are closely related activities in developing axons (Alsina et al., 2001; Fukunishi et al., 2011). Neurotransmitter release is important for the maturation of synaptic connections (Waites et al., 2005) and is unarguably affected by AP180C and mSyt1 (**Figure 4B**; Mackler et al., 2002; Nishiki and Augustine, 2004,). However, *de novo* formation of synapses does not require neurotransmission (Waites et al., 2005), and it is the appearance of new synapses that seems to be relevant for BDNF induced axon branching (Alsina et al., 2001). So even though exocytosis cannot be excluded, endocytosis appears to be a more likely mechanism.

## Conflict of Interest Statement

The authors declare that the research was conducted in the absence of any commercial or financial relationships that could be construed as a potential conflict of interest.
